# Conformational switching of ethano-bridged Cu,H_2_-bis-porphyrin induced by aromatic amines

**DOI:** 10.3762/bjnano.6.221

**Published:** 2015-11-17

**Authors:** Simona Bettini, Emanuela Maglie, Rosanna Pagano, Victor Borovkov, Yoshihisa Inoue, Ludovico Valli, Gabriele Giancane

**Affiliations:** 1 Department of Biological and Environmental Sciences and Technologies, DISTEBA, University of Salento, Via per Arnesano, Lecce, Italy; 2 Department of Engineering for Innovation, University of Salento, Via per Arnesano, Lecce, Italy; 3 Tallinn University of Technology, Faculty of Science, Department of Chemistry, Chair of Green Chemistry, Akadeemia tee 15, 12618 Tallinn, Estonia; 4 Department of Applied Chemistry, Osaka University, 2-1 Yamada-oka, Suita 565-0871, Japan; 5 Department of Cultural Heritage, University of Salento, Via per Arnesano, Lecce, Italy

**Keywords:** aromatic amines, bis-porphyrin, conformational switching, Langmuir film, surface plasmon resonance

## Abstract

Cu,H_2_-bis-porphyrin (Cu,H_2_-Por_2_), in which copper porphyrin and free-base porphyrin are linked together by an ethano-bridge, was dissolved in chloroform and spread at the air/liquid subphase interface of a Langmuir trough. The bis-porphyrin derivative, floating film was characterized by reflection spectroscopy and the surface pressure of the floating film was studied as a function of the mean area per molecule. When aromatic amines are dissolved in the subphase, an evident interaction between the bis-porphyrin host and the aromatic amine guest is observed. A clear-cut variation of the profile of surface pressure vs area per molecule curve is observed. Reflection spectroscopy highlights that the aromatic amines dissolved in the subphase are able to induce the *syn*-to-*anti* conformational switching in the bis-porphyrin derivative. The Langmuir–Schaefer technique has been used to transfer the floating bis-porphyrin film (when using pure water as a subphase) to a surface plasmon resonance (SPR) substrate and the resulting device was able to detect the presence of aniline at concentrations as low as 1 nM in aqueous solution. The high selectivity of the SPR sensing device has been verified by checking the spectral response of the active layer towards other analytes dissolved in the aqueous solutions.

## Introduction

Various porphyrin derivatives, both free-base and metal complexes, have been widely employed as active molecules for detecting analytes in vapor as well as in liquid phase [1–3]. Porphyrins are endowed with good host material properties and the ability to form films [4], allowing realization of thin film devices with variable physical and chemical properties upon complexation with guest molecules [5–7].

**Figure 1:**
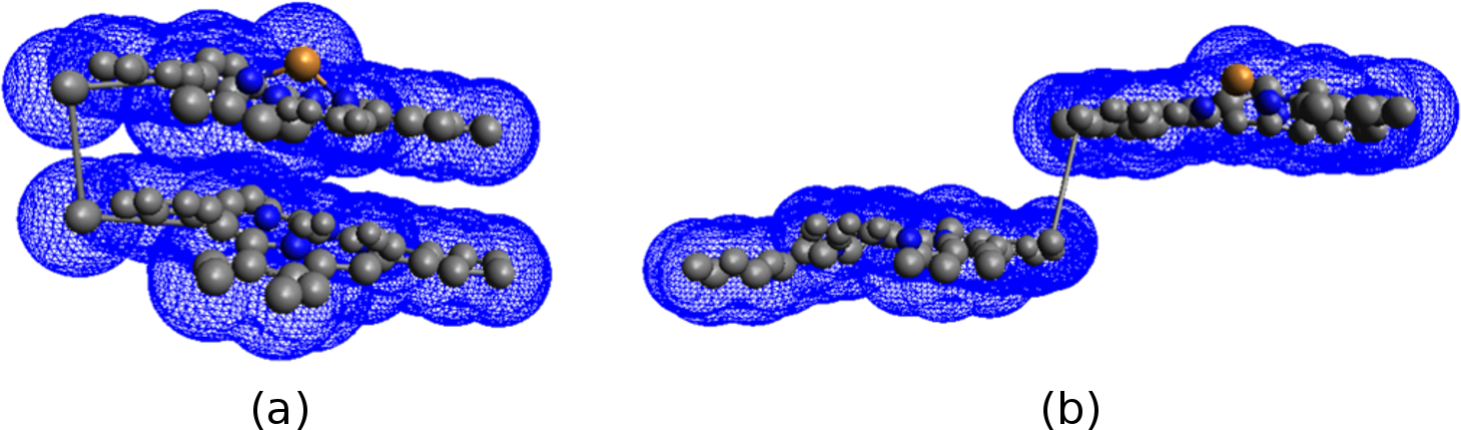
(a) *Syn*- and (b) *anti*-conformations of an ethano-bridged bis-porphyrin.

One of the most appealing classes of porphyrins is the bis-porphyrins, which can switch their conformational form as a result of the interaction with specific guest molecules, such as analytes [8]. For example, in the case of ethano-bridged bis-porphyrins, the structural change between the closed form (*syn*-form, Figure 1a) and the open form (*anti*-form, Figure 1b) can be easily detected by various spectroscopic methods.

Previously it was shown that doubly metallated bis-porphyrins transferred onto solid substrates have been effectively used to detect the presence of amines in aqueous solution [9]. The organization and orientation of active molecules in corresponding host–guest interactions play the crucial role of quenching or enhancing the affinity of a host towards a specific guest. This is particularly relevant when a structural change can be influenced by environmental conditions [10]. For this reason, a horizontal variation of the Langmuir–Blodgett technique, the Langmuir–Schaefer (LS) method, is used to transfer the active layers onto solid supports [11].

**Figure 2:**
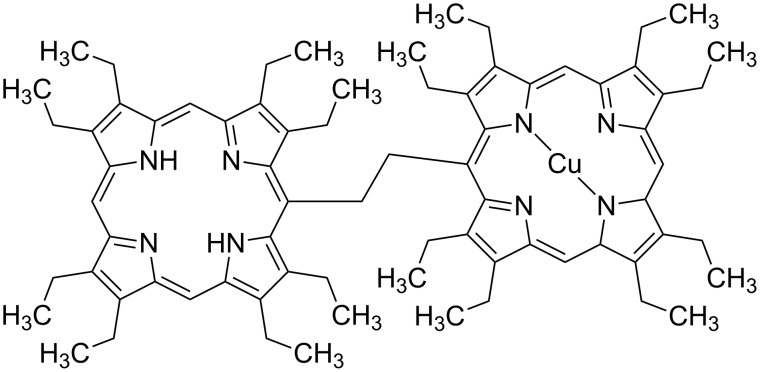
Chemical structure of the monometallated copper complex of the ethano-bridged bis-porphyrin derivative, Cu,H_2_-Por_2_.

In general, procedures to detect and remove amines in water and food matrices have been established [12–13]. Amines can be harmful to living organisms and can induce pseudo-poisoning effects, such as the scombroid syndrome [14], and in some cases, they may react with other compounds in the human body promoting the formation of cancer cells [15]. Aromatic amine sensors with different transduction methods have also been developed [16–19]. In the present work, a copper, free-base bis-porphyrin complex of ethano-bridged bis-porphyrin (shown in the Figure 2), herewith named Cu,H_2_-Por_2_, was characterized at the air/water interface and transferred by means of the LS method onto a gold SPR substrate for the detection of aromatic amines in water.

## Results and Discussion

### Air/liquid interface characterization

**Figure 3:**
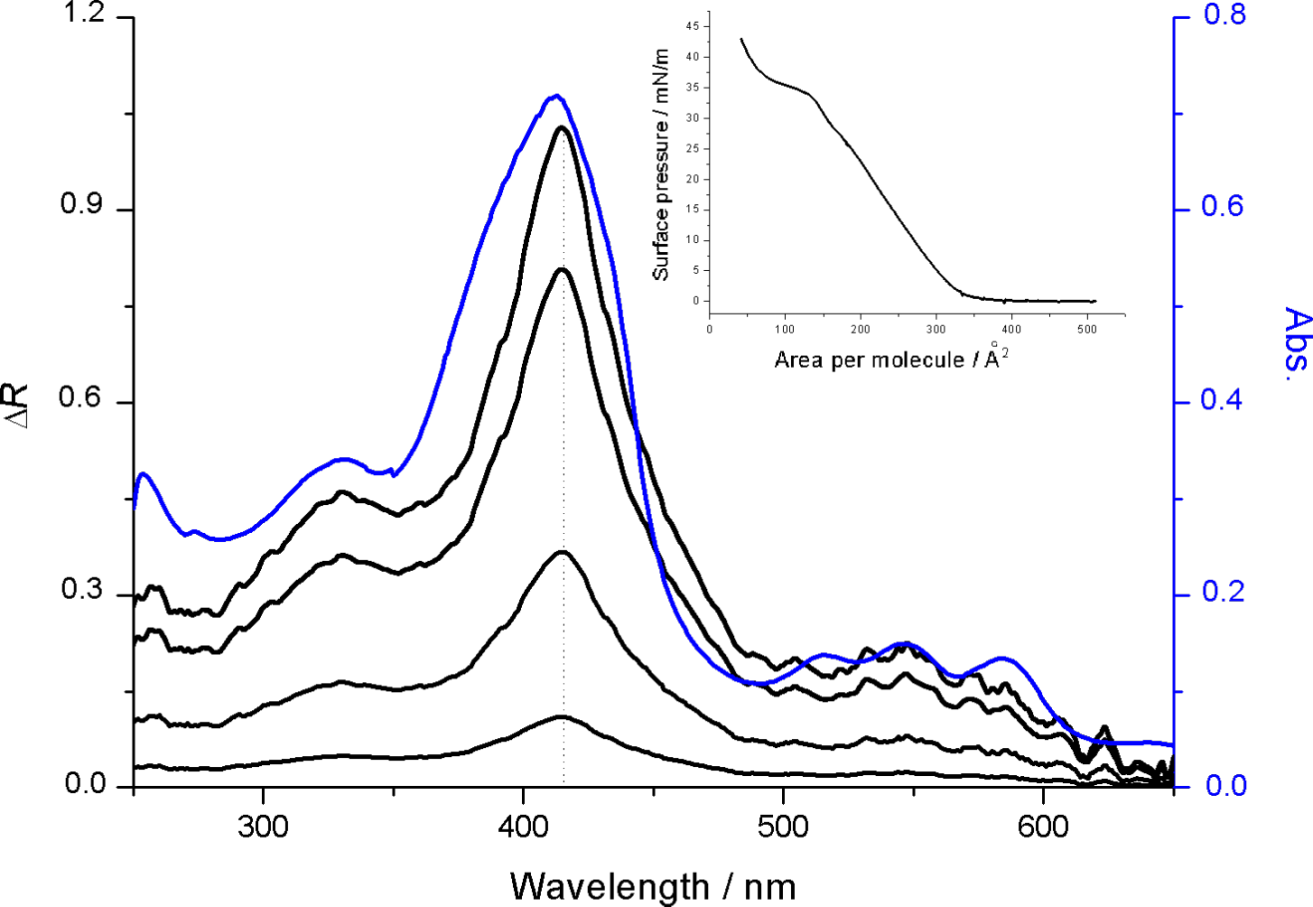
UV–vis solution spectrum (blue line) and reflection spectra of the Cu,H_2_-Por_2_ Langmuir film (black lines). The maximum reflection peak is centered at 414 nm for all the investigated surface pressures (2, 16, 35 and 40 mN/m), suggesting a closed form of the bis-porphyrin derivative as the major conformer. In the inset, the surface pressure vs area per molecule curve is reported.

Cu,H_2_-Por_2_ was dissolved in chloroform at a concentration of 1.3·10^−4^ M and the UV–vis spectrum was recorded (blue line in Figure 3). The absorption spectrum is comprised of the absorptions of the individual porphyrin moieties (Cu complex and free-base) resulting in a broadened Soret band and three peaks in the Q band region. The maximum absorption peak is centered at 414 nm, suggesting that the bis-porphyrin derivative is mainly arranged as the *syn*-conformer, whilst a minor contribution from the *anti*-form cannot be ruled out due to the flexibility of the ethano bridge [20]. After spreading 100 μL of the chloroform solution onto the ultrapure water subphase, the isotherm curve was recorded (inset of Figure 3). The surface pressure vs area per molecule curve shows at least three bends, indicating the rearrangement of the molecules in the floating film. A conformational change of the Cu,H_2_-Por_2_ molecules can be excluded upon the motion of the barriers as evidenced by the invariant absorption properties. In fact, Figure 3 (black lines) demonstrates that the reflection spectra acquired at different values of surface pressure do not show any appreciable shift of the maximum reflection peak.

**Figure 4:**
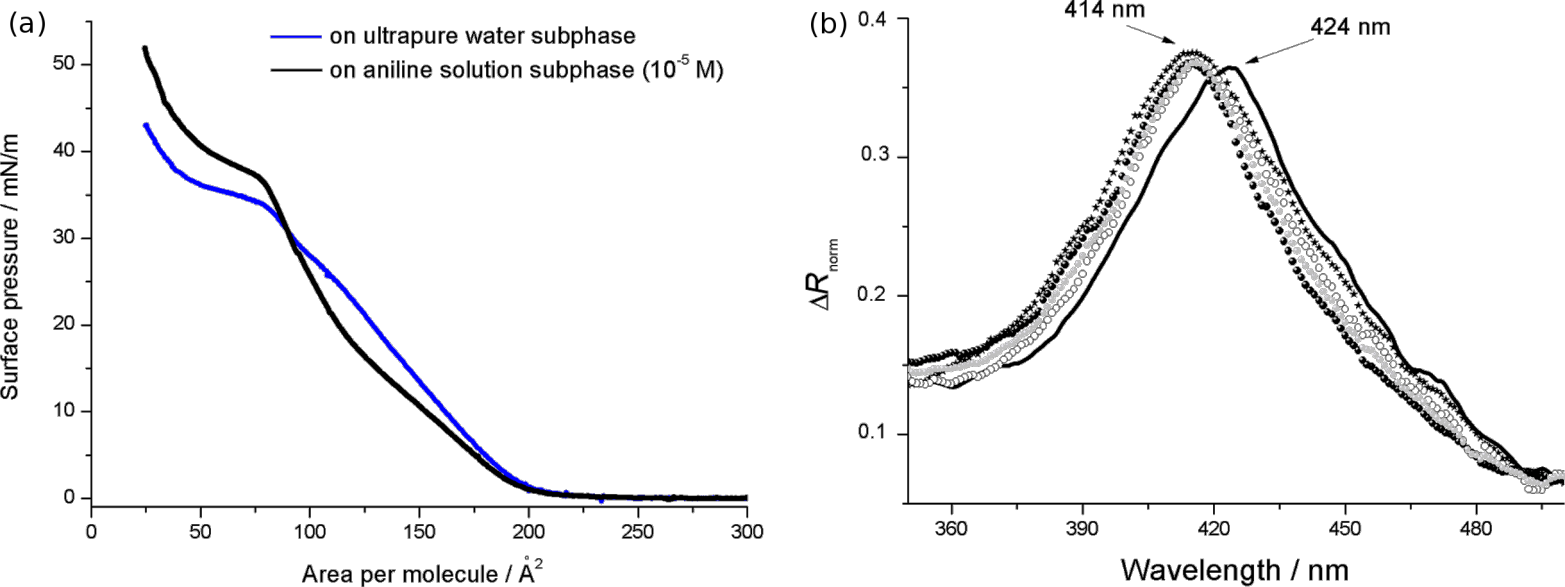
(a) The effect of aniline dissolved in the subphase (10^−5^ M) on the surface pressure vs area per molecule curve (black line) of a bis-porphyrin derivative, floating layer in comparison to the ultrapure water subphase (grey line). (b) Reflection spectra of the Cu,H_2_-Por_2_ floating film in the presence of aniline in the subphase (solid black line) in comparison with the reflection spectra of the bis-porphyrin Langmuir film spread on ultrapure water (filled black circles) and *tert*-butylamine (stars), putrescine (unfilled circles) and diaminocyclohexane (filled grey circles) aqueous solutions (all the analytes were dissolved in a concentration of 10^−5^ M). All the spectra were recorded at a surface pressure of 12 mN/m and appropriately multiplied or divided in order to have the same maximum value of Δ*R* of the Cu,H_2_-Por_2_ floating layer on the ultrapure water subphase.

The surface pressure vs area per molecule curves of Cu,H_2_-Por_2_ spread on ultrapure water and on the subphase containing aniline (10^−5^ M) have very different features (Figure 4a). Both curves show the first slope variation at a value of 200 Å^2^. At least three changes of the curve slope are recorded for the Cu,H_2_-Por_2_ on water subphase, probably a consequence of the formation of a multilayer film. The floating film spread on the subphase containing the aromatic amine showed the first slope change at about 20 mN/m and another more drastic variation at 37 mN/m. Even though the limiting area per molecule, obtained by the extrapolation of the steep region of the isotherm to zero surface pressure, is in a good agreement with a conformational change from the *anti-* to *syn-* form, such a rationale can be excluded by the reflection spectroscopy carried out at the air/water interface (Figure 4b).

Such a behavior confirms the host–guest interaction between the molecules of the floating film and the analyte dissolved in the subphase, which is further confirmed by reflection spectroscopy at the air/subphase interface. In fact, a red shift of the Soret band by about 10 nm was induced by the aniline, thus suggesting the *syn*-to-*anti* conformational switching in the Cu,H_2_-Por_2_ molecules (Figure 4b). This effect is similar to that previously observed for bis(zinc porphyrin) [8]. The behavior of the bis-porphyrin floating film upon interaction with other aliphatic acyclic and cyclic amines (*tert*-butylamine, 1,4-diaminobutane and 1,2-diaminocyclohexane) dissolved into the subphase was also checked. It was found that there are no variations in the absorption spectra, suggesting that the aromatic group of the analyte is crucial for the conformational switching in the bis-porphyrin derivative. Therefore, these results prompted us to test a phenol solution as a subphase for the Cu,H_2_-Por_2_ floating film. As highlighted by the invariant reflection spectrum (Figure 4b), the phenol guest does not induce a conformational change. Therefore, it is reasonable to suggest that the simultaneous presence of an amino group and aromatic ring is necessary to induce the *syn*-to-*anti* conformational change in Cu,H_2_-Por_2_. As a further confirmation of such rationale, for the bis-porphyrin floating film obtained on a water subphase containing 10^−5^ M 2-methyl-2-propanethiol, the *syn*-conformer remains unchanged even at high surface pressure values.

**Figure 5:**
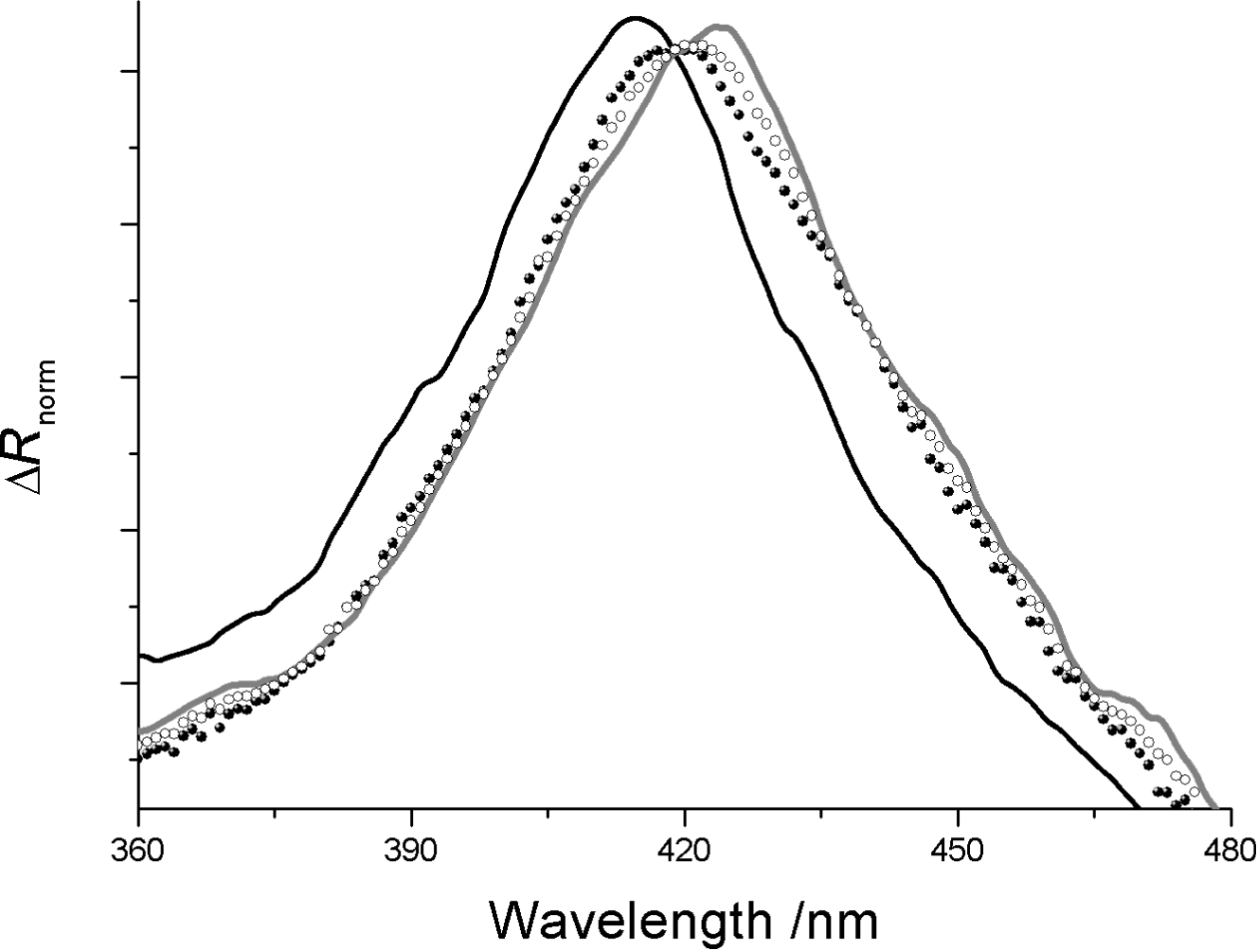
Normalized reflection spectra of the Cu,H_2_-Por_2_ floating films spread on a subphase containing *N*-methylphenethylamine (filled black circles), α-methylbenzylamine (unfilled circles) and aniline (grey solid line) (10^−5^ M) compared with the reflection spectrum of the bis-porphyrin derivative, floating film recorded on ultrapure water (black solid line). All the spectra were recorded at a surface pressure of 12 mN/m and appropriately multiplied or divided in order to have the same maximum value of Δ*R* of the Cu, H_2_-Por_2_ floating layer on the ultrapure water subphase.

In order to confirm this assumption, the effect of two additional aromatic amines on the bis-porphyrin derivative, floating film was investigated. α-Methylbenzylamine and *N*-methylphenethylamine were dissolved in the subphase at a concentration of 10^−5^ M. As was the case for aniline, α-methylbenzylamine and *N*-methylphenethylamine induced the corresponding red shift in the reflection maximum of the Cu,H_2_-Por_2_ floating film (Figure 5). It is likely that the effect of the aromatic amines is influenced by the steric hindrance of the guest molecule. A more detailed host–guest interaction mechanism will be the subject of future investigations.

### Amine sensing experiments

**Figure 6:**
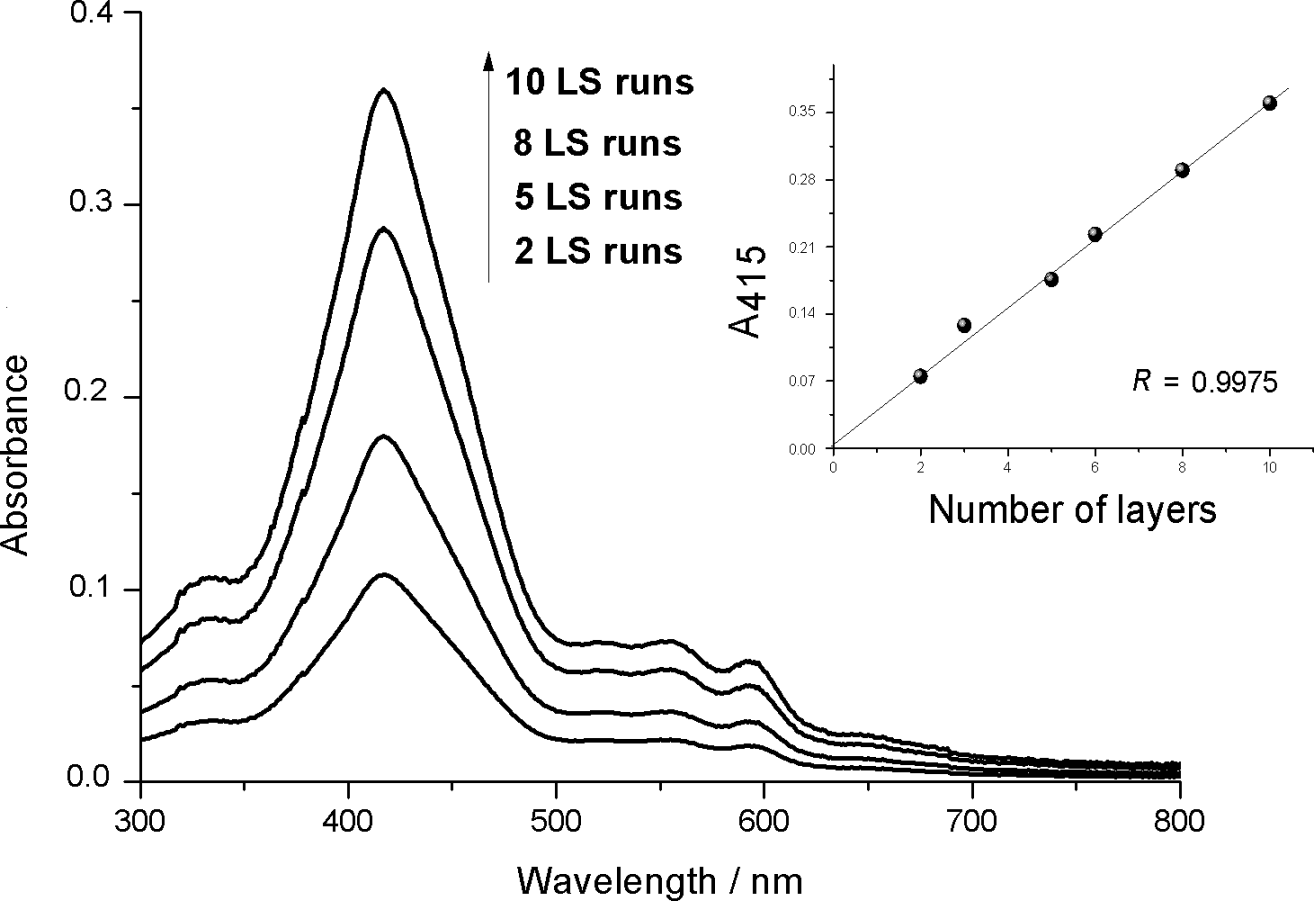
UV–vis spectra in the range of 300–800 nm of the Cu,H_2_-Por_2_ Langmuir–Schaefer films. The linear dependence of the absorbance on the LS layer number is highlighted in the inset.

In order to utilize the observed host–guest interaction between the bis-porphyrin derivative and aromatic amines, amine sensing experiments have been carried out. A Langmuir film of Cu,H_2_-Por_2_ was repeatedly transferred by the LS method onto quartz substrates and the UV–vis spectra were recorded with each additional LS run (Figure 6). For all the LS films, the maximum absorption peak was the same and centered at 414 nm suggesting that the molecular conformation was not changed with the deposition process retaining essentially the closed *syn*-form as in the case of the floating Cu,H_2_-Por_2_ Langmuir film.

The linear relationship between the number of Cu,H_2_-Por_2_ layers and the absorbance peak illustrated the good deposition rate and uniformity of the deposition procedure (Figure 6, inset). Furthermore, the absorption profile was not changed with increasing layer deposition, suggesting negligible interlayer interactions.

**Figure 7:**
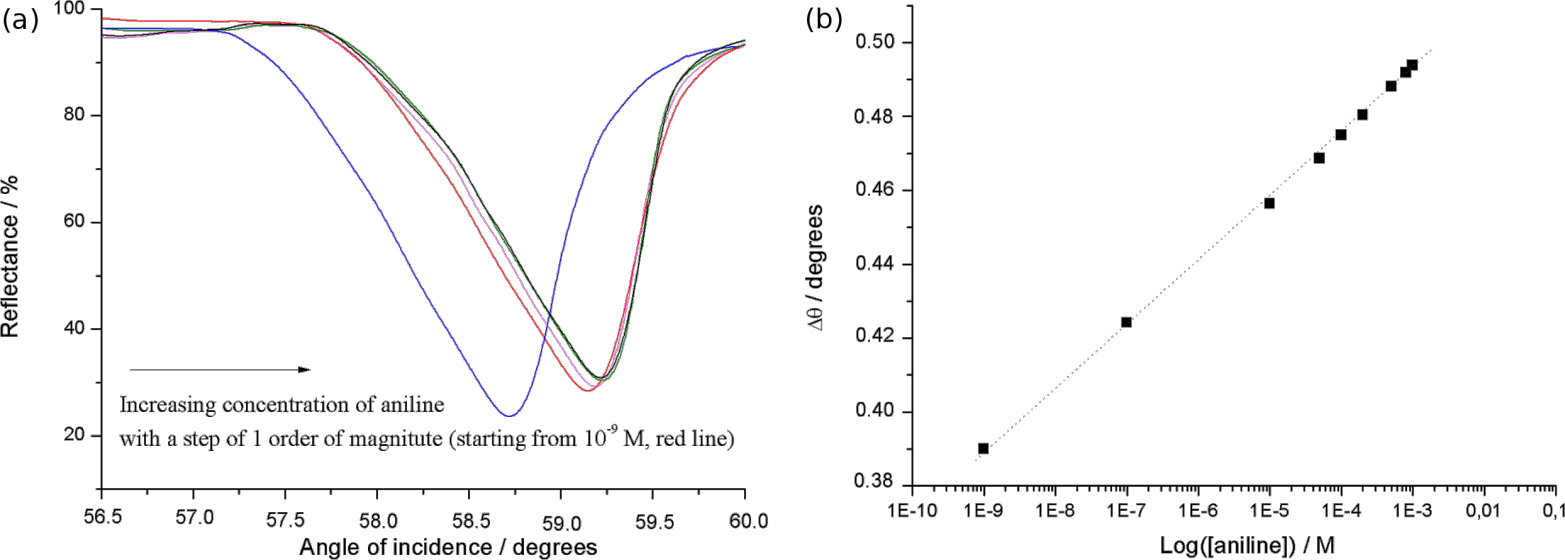
(a) Reflectance variation of Cu,H_2_-Por_2_ LS films and (b) SPR angle shift as a function of analyte concentration.

Five LS runs of Cu,H_2_-Por_2_ were deposited on the SPR slide, and the shift of the SPR angle induced by the injection of amine aqueous solutions at different concentrations was monitored. The effect of aniline on the plasmon resonance of the Cu,H_2_-Por_2_ film could be detected when only 1 nM of analyte was fluxed over the active layer (Figure 7a). A semi-logarithmic dependence of the SPR angle shift on the aniline concentration is evident at least up to 1 mM with a dynamic range of more than 6 orders of magnitude (Figure 7b). This behavior can be explained by the equation:


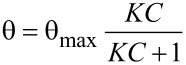


where *K* is the binding constant and *C* is the analyte concentration [21]. If the product *KC* is relatively small (<<1), the SPR angle θ is linearly dependent on the analyte concentration. On the contrary, when *KC* is comparable to 1, the linearity is not preserved. This deviation from linearity was thoroughly studied and reported in the literature and a semi-logarithmic trend was proposed [22–24].

The recovery of the SPR device was investigated. The initial plasmon resonance angle was obtained when the Cu,H_2_-Por_2_ LS film, previously exposed to aniline, was treated for 15 min at 50 °C and then washed with a flow of ultrapure water for 5 min.

**Figure 8:**
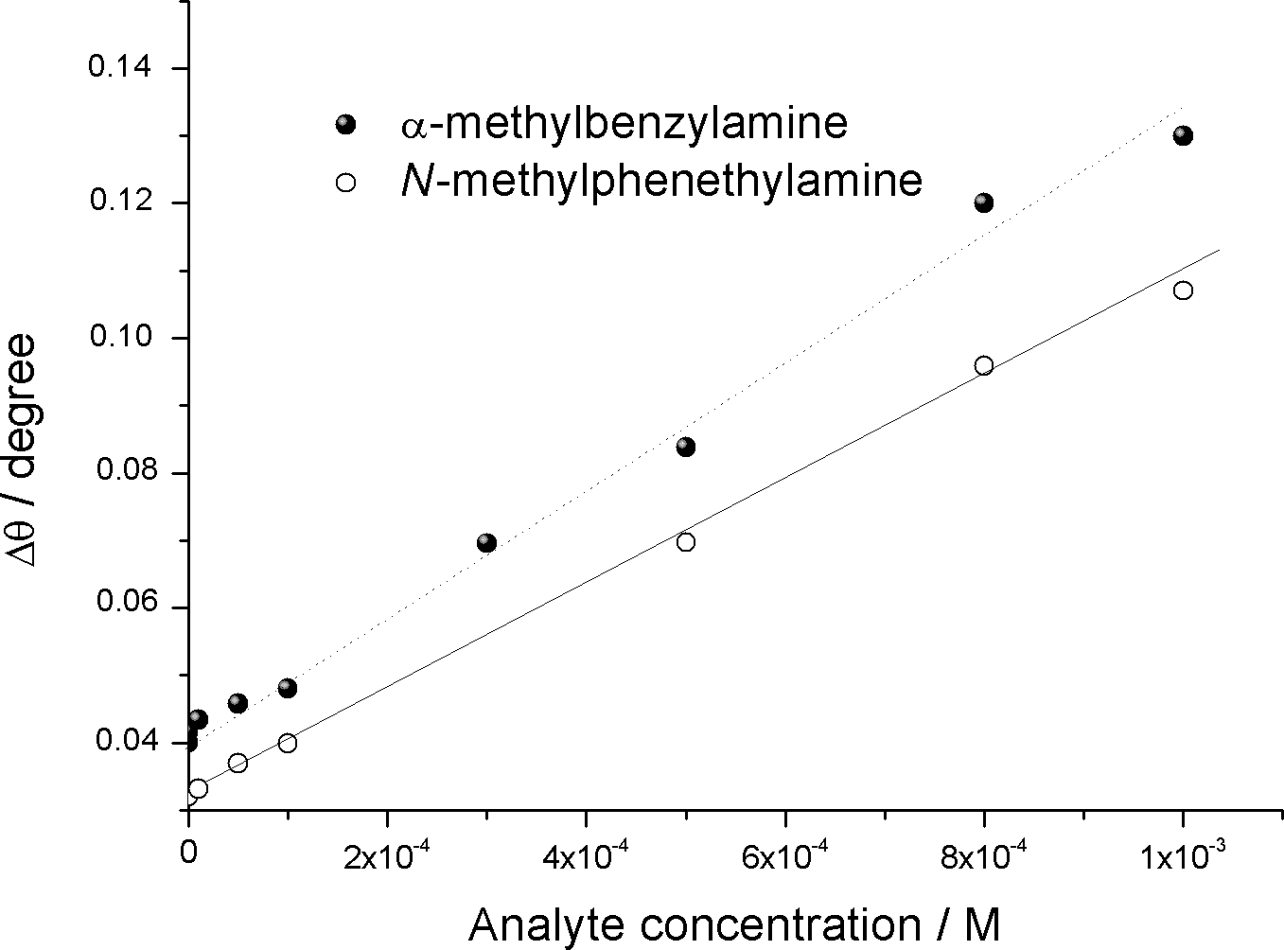
SPR angle shift as a function of α-methylbenzylamine (filled black circles) and *N*-methylphenethylamine (unfilled circles) concentration in aqueous solution.

The response of the Cu,H_2_-Por_2_ device to α-methylbenzylamine and *N*-methylphenethylamine was also monitored and a reduced sensitivity of the active layer towards these molecules was observed (Figure 8). This behavior is in good agreement with the reflection spectra recorded on the Langmuir film. α-Methylbenzylamine and *N*-methylphenethylamine induced a less intense shift of the reflection peak of the Cu,H_2_-Por_2_ Langmuir film in comparison with aniline. Both the reflection spectroscopy and SPR measurements suggested that α-methylbenzylamine and *N*-methylphenethylamine weakly interact with Cu,H_2_-Por_2_ molecules. However, the injection of phenol solutions (up to 0.01 M) did not induce any detectable shift in the plasmon peak of the SPR sample.

## Conclusion

In this study, an ethano-bridged bis-porphyrin with a free-base ring and a copper metallated ring was dissolved in a chloroform solution and spread at the air/liquid interface of a Langmuir trough. The floating film was characterized both on the ultrapure water subphase and on the subphase containing different amine solutions. The surface pressure vs area per molecule curve was influenced by the presence of aromatic amines, and in particular aniline, in the subphase. A confirmation of the host–guest interaction between the floating molecule and the analyte in the subphase was provided by the reflection spectroscopy measurements carried out directly at the air/subphase interface. This interaction induces the *syn*-to-*anti* conformation switching in the structure of the bis-porphyrin, hence producing an approximately 10 nm red shift of the absorption maximum. Neither aliphatic amines nor phenol induced the same variation in the reflection spectra of the Langmuir film, suggesting that a cooperative effect of the amine and aromatic groups is needed. The bis-porphyrin Langmuir film was transferred onto an SPR substrate and the host–guest interaction with amines in aqueous solution was investigated. Interestingly, the preliminary sensor tests evidenced that a significant angle shift of the surface plasmon resonance was recorded when only 1 nM of aniline was fluxed on the active layer. According to the reflection spectroscopy results, this interaction appeared to be highly selective towards aniline and more general towards aromatic amines, proposing the Cu,H_2_-bis-porphyrin derivative as an effective active layer for aromatic, amine sensors in aqueous solution.

## Experimental

Cu,H_2_-Por_2_ was synthesized by a previously reported method [20].

A NIMA trough equipped with two optical fibers was used for the reflection spectroscopy measurements and the same trough was employed for transferring the LS films. A chloroform solution of Cu,H_2_-Por_2_ was spread onto the aqueous subphase and the floating film formed was left to stand for 15 min before starting the Langmuir experiment. A barrier speed of 5 mm/min was used in all the experiments at the air/water interface. The reflection spectra were obtained as a difference between the reflectivities of the clean subphase and the Cu,H_2_-Por_2_ floating film.

Aqueous solutions of amines were fluxed over the LS, Cu,H_2_-Por_2_ films deposited on metal/glass substrates (Corning 7059, with a refractive index of 1.723 at 632.8 nm, gold thickness of 44 nm) by a peristaltic pump. The SPR measurements were carried out using a Nanofilm apparatus.
